# Psychiatrists' Attitudes toward Metabolic Adverse Events in Patients with Schizophrenia

**DOI:** 10.1371/journal.pone.0086826

**Published:** 2014-01-23

**Authors:** Norio Sugawara, Norio Yasui-Furukori, Manabu Yamazaki, Kazutaka Shimoda, Takao Mori, Takuro Sugai, Yutaro Suzuki, Toshiyuki Someya

**Affiliations:** 1 Department of Neuropsychiatry, Hirosaki University School of Medicine, Hirosaki, Japan; 2 Japanese Society of Clinical Neuropsychopharmacology, Tokyo, Japan; 3 Japan Psychiatric Hospitals Association, Tokyo, Japan; 4 Department of Psychiatry, Dokkyo Medical University School of Medicine, Mibu, Japan; 5 Department of Psychiatry, Niigata University Graduate School of Medical and Dental Sciences, Niigata, Japan; Chiba University Center for Forensic Mental Health, Japan

## Abstract

**Background:**

There is growing concern about the metabolic abnormalities in patients with schizophrenia.

**Aims:**

The aim of this study was to assess the attitudes of psychiatrists toward metabolic adverse events in patients with schizophrenia.

**Method:**

A brief questionnaire was constructed to cover the following broad areas: the psychiatrists' recognition of the metabolic risk of antipsychotic therapy, pattern of monitoring patients for physical risks, practice pattern for physical risks, and knowledge of metabolic disturbance. In March 2012, the questionnaire was mailed to 8,482 psychiatrists who were working at hospitals belonging to the Japan Psychiatric Hospitals Association.

**Results:**

The overall response rate was 2,583/8,482 (30.5%). Of the respondents, 85.2% (2,200/2,581) reported that they were concerned about prescribing antipsychotics that have a risk of elevating blood sugar; 47.6% (1,201/2,524) stated that their frequency of monitoring patients under antipsychotic treatment was based on their own experiences; and only 20.6% (5,22/2,534) of respondents answered that the frequency with which they monitored their patients was sufficient to reduce the metabolic risks.

**Conclusions:**

Psychiatrists practicing in Japan were generally aware and concerned about the metabolic risks for patients being treated with antipsychotics. Although psychiatrists should monitor their patients for metabolic abnormalities to balance these risks, a limited number of psychiatrists answered that the frequency with which they monitored patients to reduce the metabolic risks was sufficient. Promotion of the best practices of pharmacotherapy and monitoring is needed for psychiatrists treating patients with schizophrenia.

## Introduction

Schizophrenia is a severe mental illness that affects approximately 1% of the population worldwide [1]. Recently, a high prevalence of metabolic syndrome (MetS) has been reported among patients with schizophrenia [2]–[4]. MetS has been related to an increased risk for cardiovascular diseases [5], [6], diabetes [7] and mortality [8]; it is defined as a cluster of metabolic disturbances, including abdominal obesity, atherogenic dyslipidemia, hypertension, and hyperglycemia [9].

Although atypical antipsychotics have greatly improved extrapyramidal side effects in patients with schizophrenia, weight gain [10], increased levels of fasting glucose and lipids [11] and dysregulation of adipocytokines [12] have been reported during treatment with some atypical antipsychotics. In particular, some atypical antipsychotics, such as olanzapine and clozapine, have been consistently demonstrated to promote insulin resistance and dyslipidemia [13], [14].

Psychiatrists’ attitudes toward metabolic issues, is a relatively unexplored factor that may be important. Educational program for psychiatrists might be an effective means of improving their clinical behavior or patient outcomes [15]–[17]. To date, a limited number of studies [18]–[20] have assessed psychiatrists’ attitudes toward metabolic issues among patients under antipsychotic treatment. However, those studies had relatively small sample sizes and low response rates. Thus, it is necessary to accurately assess psychiatrists’ attitudes toward metabolic issues in a representative sample of psychiatrists.

In this study, we investigated psychiatrists’ attitudes toward metabolic adverse events in a nationwide survey of Japanese psychiatrists. To our knowledge, this is the largest study of psychiatrists and the first investigation involving the attitudes of Asian psychiatrists toward metabolic issues.

## Method

### Ethics Statement

All respondents provided their verbal informed consent to participate in this study without any incentive. The anonymous questionnaire was the only research instrument and a statement was included that says "Completion of the attached questionnaire will be taken as indicating your consent to participate". The study procedure was approved by the Ethics Committee at the Japan Psychiatric Hospitals Association and the Hirosaki University School of Medicine.

### Procedure

This survey was prepared by the joint committee of Japanese Society of Clinical Neuropsychopharmacology and Japan Psychiatric Hospitals Association for antipsychotics treatment and physical risk. After reviewing the relevant literature and extant guidelines, a brief questionnaire was constructed to cover the following broad areas: the psychiatrists' recognition of metabolic risk with antipsychotic therapy, their pattern of monitoring patients for physical risks, their practice pattern in physical risks, and their knowledge of metabolic disturbance (see appendix S1). In March 2012, the questionnaire was mailed to hospitals belonging to the Japan Psychiatric Hospitals Association. The survey was completed by October 2012. According to the latest data provided by the Japanese Ministry of Health, Labour and Welfare, the total number of psychiatrists in Japan is 14,201. Psychiatrists working at university hospital or private clinic were not included in this survey.

## Results

**Figure 1:**
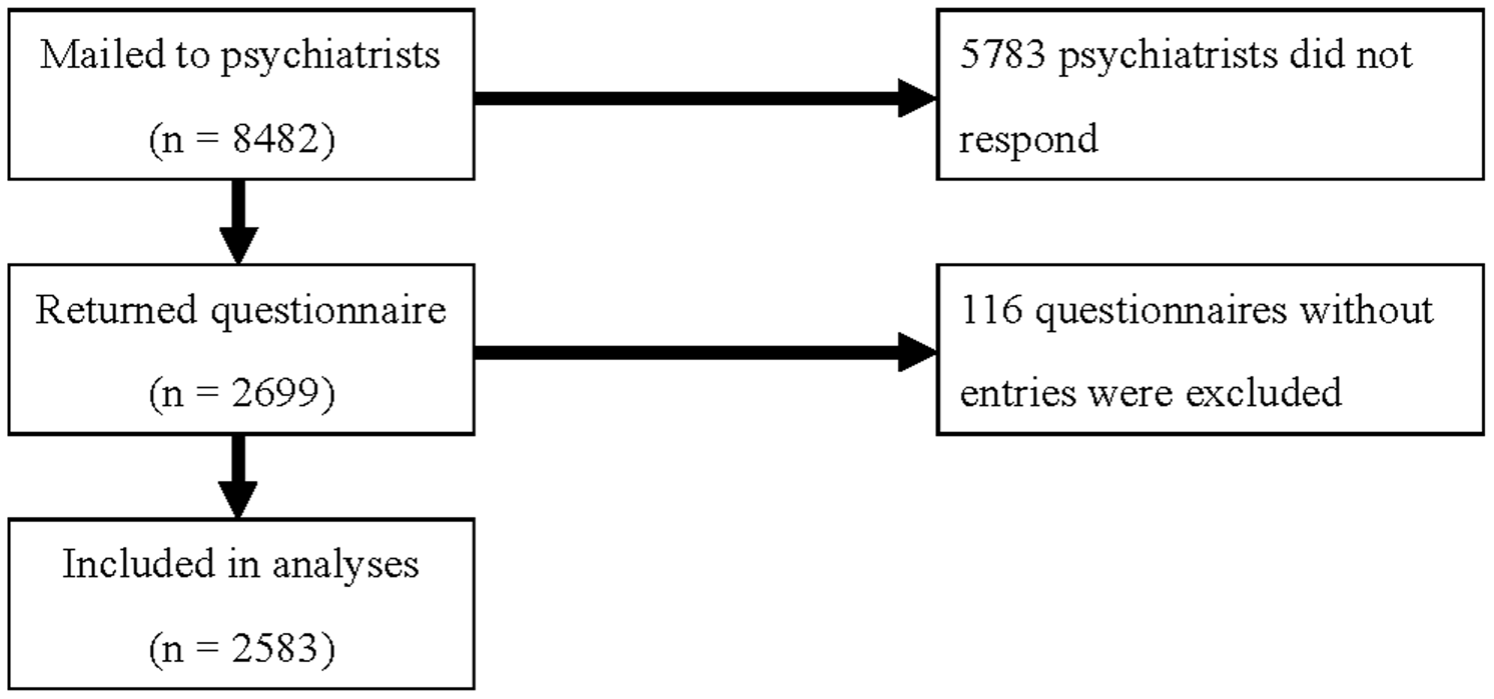
Recruitment and Participant Flow Chart.

As shown in Figure 1, we mailed a questionnaire to 8,482 psychiatrists who were working at hospitals belonging to the Japan Psychiatric Hospitals Association. Among 2,699 psychiatrists sending the questionnaire back, 116 respondents turned in blank paper. The overall response rate was 30.5% (2,583/8,482). The demographic characteristics of the study subjects are listed in Table 1.

**Table 1:** Characteristics of study subjects (N  =  2583).

Variables		
Age (years)	48.8±13.0	
<39	738	(28.6)
40 and over	1845	(71.4)
Gender		
Male	2039	(78.9)
Female	544	(21.1)
Clinical experience in psychiatry (years) year	19.2±13.2	

### Psychiatrists’ recognition of metabolic risk with antipsychotic therapy

In this survey, 13.7% of respondents (350/2,552) experienced a patient with a rapid elevation in their blood glucose within the past 6 months (Q7). Of the respondents, 85.2% (2,200/2,581) reported that, with concern, they prescribe antipsychotics that have a risk of elevating blood sugar, and 14.8% (381/2,581) of the respondents did not have such a concern (Q4). Among psychiatrists answering "No" to Q4, 52.8% (201/381) of respondents described the reason "I administer weight control and nutritional intervention", 21.5% (82/381) of respondents described "I have never experienced any blood glucose abnormalities", and 13.1% (50/381) of respondents described "I routinely monitor my patients" (Q5). In response to an informed consent discussion with their patients about the adverse effects of elevated blood glucose, 70.9% (1,789/2,522) of the respondents explained the risk (orally 69.7%, with documentation 1.2%), 24.1% (609/2,522) explained it only to patients with personal or family histories of diabetes, and 4.9% (124/2,522) did not explain the risk (Q6).

### Pattern of monitoring patients for physical risks

With regard to monitoring their patients’ body weights, 25.3% (622/2,461) of the respondents focused on "patients taking drugs with a risk of elevated blood glucose and weight gain", 24.4% (601/2,461) of respondents focused on "all patients", 21.0% (517/2,461) of respondents answered "not routinely; only when I notice", 16.8% (414/2,461) of respondents focused on "obese patients", 11.1% (274/2,461) of respondents focused on "patients with a personal or family histories of diabetes" and 1.3% (33/2,461) of respondents did not focus on specific kind of patients (Q9). Among the psychiatrists who answered except "none" for Q9, the respondents reported monitoring their inpatients’ weights at the following intervals: once a week 17.8% (289/1,620), once a month 76.3% (1,236/1,620), twice a year 5.2% (84/1,620), and once a year 0.7% (11/1,620). The respondents also reported monitoring their outpatients’ weights at the following intervals: every visit 29.1% (413/1,419), once every three months 24.6% (349/1,419), twice a year 24.4% (346/1,419) and once a year 21.9% (311/1,419) (Q10). In this survey, only 4.5% (116/2,583) of the respondents answered that they routinely monitored the waist circumference of patients under antipsychotic treatment (Q13). Table 2 shows the patterns of monitoring patients for other metabolic risks during antipsychotic treatment (Q13, Q14).

**Table 2:** Pattern of monitoring patients for weight gain and metabolic risks during antipsychotic treatment.

	Fasting blood glucose	Hemoglobin A1c	Dietary habit	Intake of soft drinks	Blood pressure	Lipid profile	Electro-cardiography
**Inpatients**							
more than once a month	28.6 (697/2434)	14.7 (361/2453)	36.1 (889/2462)	24.6 (610/2480)	48.9 (1206/2468)	26.2 (645/2460)	6.8 (171/2522)
more than twice a year	41.5 (1010/2434)	31.2 (765/2453)	16.2 (399/2462)	10.9 (271/2480)	9.5 (235/2468)	37.6 (924/2460)	20.6 (520/2522)
once a year	6.2 (151/2434)	7.3 (178/2453)	2.8 (70/2462)	2.1 (51/2480)	1.2 (30/2468)	4.6 (114/2460)	8.6 (216/2522)
not routinely	23.7 (576/2434)	46.8 (1149/2453)	44.8 (1104/2462)	62.4 (1548/2480)	40.4 (997/2468)	31.6 (777/2460)	64.0 (1615/2522)
**Outpatients**							
more than once a month	3.9 (93/2406)	2.7 (67/2440)	20.5 (517/2526)	14.0 (346/2474)	24.0 (574/2395)	3.9 (95/2423)	1.0 (23/2420)
more than twice a year	48.2 (1159/2406)	31.6 (771/2440)	25.5 (645/2526)	17.3 (427/2474)	23.8 (569/2395)	42.7 (1035/2423)	11.9 (287/2420)
once a year	23.3 (560/2406)	17.9 (436/2440)	6.9 (174/2526)	5.7 (142/2474)	9.3 (222/2395)	20.8 (503/2423)	15.2 (367/2420)
not routinely	24.7 (594/2406)	47.8 (1166/2440)	47.1 (1190/2526)	63.0 (1559/2474)	43.0 (1030/2395)	32.6 (790/2423)	72.0 (1743/2420)

In addition, 47.6% (1,201/2,524) of the psychiatrists stated that their frequency of monitoring patients under antipsychotic treatment was based on their own clinical experience, 14.5% (367/2,524) stated that it was based on guidelines, and 10.1% (254/2,524) stated that it was based on the guideline advice of specialists, while 27.8% (702/2,524) answered that they did not base it on specific principles (Q15). However, only 20.6% (522/2,534) of the respondents answered that their frequency of monitoring patients to reduce the metabolic risk was sufficient, while 79.4% (2,012/2,534) of respondents answered “I don’t know” or ”Not sufficient” (Q17).

### Practice pattern in physical risks

In response to detecting adverse metabolic effects, such as elevated blood glucose or dyslipidemia, 80.2% (2,000/2,494) of the respondents stated that they would switch to a different antipsychotic, and 14.7% (366/2,494) of the respondents stated that they would consult with specialists (Q8). In response to treating patients with stable mental conditions under antipsychotic treatment who showed weight gain, 55.0% (1,396/2,537) of the respondents stated that they would observe the course of the patients with monitoring, 25.9% (657/2,537) of the respondents stated that they would switch to a different antipsychotic, and 12.1% (307/2,537) of the respondents stated that they would switch to a lower dose of the current medication (Q11). With regard to having an opportunity to consult with diabetes specialists, 71.4% (1,822/2,551) of the respondent answered that they were able to contact a specialist if necessary, 21.2% (542/2,551) of the respondents answered that they usually contacted a specialist, and 7.3% (187/2,551) of the respondents answered that they could not contact a specialist (Q18).

### Knowledge of metabolic disturbance

When asked whether they referred to the definition of metabolic syndrome in the clinical setting, only 3.4% (87/2,570) of the respondents answered that they usually used the definition, 32.8% (847/2,570) of the respondents answered that they partially used the definition, 31.4% (808/2,570) of the respondents answered that they used the definition in some cases, and 32.2% (828/2,570) of the respondents answered that they did not use the definition at all (Q12).

With regard to the Homeostasis model assessment-Insulin Resistance (HOMA-IR), 89.2% (2,273/2,547) of the respondents answered that they did not use the definition at all, 10.4% (264/2,547) of the respondents answered that they used the definition in some cases and 0.4% (10/2,547) of the respondents answered that they usually used the definition (Q16).

With regard to blood pressure control criteria, 12.1% (309/2,547) of the respondents answered that they used the criteria of 130/85, 61.5% (1,567/2,547) of the respondents answered that they used the criteria of 140/90 and 25.0% (638/2,547) answered that they used the criteria of 160/100 (Q19).

## Discussion

The present study on the attitudes toward metabolic adverse events in patients with schizophrenia is a national survey of psychiatrists in Japan. In our survey, 85.2% of the respondents reported that they prescribed antipsychotics with a risk of elevating blood sugar with concern, and the majority (80.2%) stated that they would switch to a different antipsychotic in response to detecting an adverse metabolic effect. Our results confirm that the metabolic adverse events among patients under antipsychotic treatment are now considered to be substantial and compelling adverse effects that alter psychiatrists' prescribing of these medications.

Our results concerning the recognition of metabolic adverse events in patients with schizophrenia are similar to those reported in previous reports. In the Atypical Antipsychotic Therapy and Metabolic Issues (AtAMI) survey [18], the overwhelming majority of psychiatrists (97%) seriously considered metabolic abnormalities. Another study conducted by Buckley et al. [19] showed that 82% of psychiatrists thought that the risk for such abnormalities among their patients who were receiving antipsychotic medications was higher than in the general population. With regard to discussing informed consent, our study showed that only a minority of psychiatrists (1.2%) actually used a document to explain the risk of elevated blood glucose to patients when they were taking antipsychotics, similar to the results of Buckley et al. [19].

Regarding the monitoring of waist circumference, our respondents answered that waist circumference measurements were infrequently obtained (4.5%). The same pattern was observed in previous studies. The low performance for measuring the waist circumference of patients might be because of psychiatrists' unease that the measurement of this parameter is inappropriate [19]. The waist circumference is a useful monitoring parameter that can be used as a quick and simple way to identify patients who are susceptible to MetS [21]. Educational efforts to increase the awareness of appropriate monitoring in clinical practice are needed for psychiatrists.

With respect to the monitoring of lipid profiles, blood glucose, and blood pressure for patients, Buckley et al. [19] showed that majority of the respondents did not routinely monitor lipid profiles, blood glucose, and blood pressure. In our survey, the majority of respondents reported that they monitor lipid profiles and blood glucose more than twice a year among both inpatients and outpatients. As to monitoring blood pressure, our results showed that inpatients were monitored more frequently than outpatients. This discrepancy may be related to differences in the insurance system and in attitudes toward patient monitoring.

Consistent with previous results [19], the majority of the respondents (80.2%) indicated that adverse metabolic effects had altered their prescribing of antipsychotics in their practice. The AtAMI survey [18], which assessed practice patterns in detail, showed that 83% of the respondents said that they would avoid certain atypical antipsychotics for patients with diabetes or a family history of diabetes, 80% said that they would do so in patients who are obese or who have a high body mass index, and 77% said that they would avoid certain atypical antipsychotics in patients with a cardiovascular condition or family histories of cardiovascular disease.

In our survey, the majority of the respondents stated that their frequency of monitoring patients under antipsychotic treatment was based on their own clinical experiences. However, only 20.6% of the respondents answered that their frequency of monitoring the patients to reduce the metabolic risk was sufficient. It is necessary for psychiatrists to acquire knowledge in this area and refer to recent medical reports to reduce the risk of metabolic adverse events among patients with schizophrenia.

The present study included 8,482 psychiatrists, all of whom were sent the survey. However, only 2,583 completed the questionnaire, yielding a response rate of 30.5%. It is plausible that psychiatrists who are more interested and have referred to the emergent literature regarding metabolic side effects may have been more likely to respond compared to other psychiatrists; therefore, the participants of this study might not necessarily be representative of all psychiatrists. Although this study is the largest involving psychiatrists and the first investigation assessing the attitudes toward metabolic adverse events in non-western countries, we cannot completely rule out the selection bias of our participants. In addition, Japan has the highest number of psychiatric beds per 100,000 people in the world [22]. The mean length of hospital stay is about 1.5 years [23]. Domestic situation in Japan might affect the attitudes of psychiatrists in this study [24]. Furthermore, our study is limited by the fact that it is cross-sectional rather than prospective in design and lacks data on the actual practices of psychiatrists. This study could not clarify a causal relationship between the psychiatrists' recognitions or knowledge and any effect on their practice in the clinical setting. Future studies with longitudinal designs and data concerning the actual practices are needed to investigate these associations.

This study demonstrates that psychiatrists practicing in Japan were generally aware and concerned about the metabolic risks for patients treated with antipsychotics. Although psychiatrists should monitor patients for metabolic abnormalities to balance these risks, a limited number of psychiatrists answered that their frequency of monitoring patients to reduce the metabolic risk was sufficient. Educational efforts [25] and promoting the best practices of pharmacotherapy and monitoring [26] will be needed for psychiatrists treating patients with schizophrenia.

## Supporting Information

Appendix S1 Questionnaire about psychiatrists' attitudes toward metabolic adverse events in patients with schizophrenia.
(DOC)
