# Supplementary material for: Psychiatrists' Attitudes toward Metabolic Adverse Events in Patients with Schizophrenia
Source: PLoS One. 2014 Jan 23;9(1):e86826. doi: 10.1371/journal.pone.0086826 (PMC3900677; doi:10.1371/journal.pone.0086826)
Supplement: Appendix S1 — Questionnaire about psychiatrists' attitudes toward metabolic adverse events in patients with schizophrenia. (DOC) [file pone.0086826.s001.doc]

**Appendix S1**

Q1 Please write your age and sex.

[ ] years old male / female

Q2 How long have you worked in the present hospital?

[ ] years

Q3 How long have you had clinical experience in psychiatry?

[ ] years

Q4 Have you been concerned when prescribing antipsychotics with a risk of elevating blood sugar levels?

Yes / No

Q5 If you answered "No" to Q4, then why have you not been concerned with prescribing an antipsychotic with a risk of elevating blood sugar?

Yes / No I have never experienced any blood glucose abnormalities.

Yes / No I routinely monitor my patients.

Yes / No I administer weight control and nutritional intervention.

Yes / No Other

Q6 How do you explain the risks of antipsychotics with the adverse effect of elevating blood glucose?

□ orally

□ with a document

□ explain only to patients with a personal or family history of diabetes

□ do not explain the risk

Q7 Have you experienced a patient with a rapid elevation in their blood glucose within the past 6 months?

Yes / No

Q8 How do you respond to the detection of adverse metabolic effects, such as elevated blood glucose or dyslipidemia?

□ do not respond

□ switch to a different antipsychotic

□ consult with specialists

□ other

Q9 What kind of patient do you focus on by monitoring their body weight?

□ all patients

□ patients taking drugs with a risk of elevated blood glucose and weight gain

□ patients having a personal or family history of diabetes

□ obese patients

□ not routinely; only when I notice

□ none

Q10 To those who answered except "none" for Q9, how often do you monitor your patients' body weight?

For inpatients

□ once a week □once a month □ twice a year □ once a year

For outpatients

□ every visit □ once every three months □ twice a year □ once a year

Q11 How do you respond to the detection of patients who showed weight gain with a stable mental condition under antipsychotic treatment?

□ observe course of patients with monitoring

□ switch to a different antipsychotic

□ switch to a lower dose of the current medication

□ other

Q12 How do you refer to the definition of a metabolic syndrome in the clinical setting?

□ usually use the definition

□ partially use the definition

□ use the definition in some cases

□ do not use the definition at all

Q13 What kind of monitoring, except for body weight, do you routinely carry out among patients under antipsychotic treatment? (question with open-ended choices)

□ Waist circumference

□ Fasting blood glucose

□ Hemoglobin A1c

□ Dietary habit

□ Intake of soft drinks

□ Blood pressure

□ Lipid profile

□ Electrocardiography

Q14 About items selected in Q13

How often do you monitor your patients?

For inpatients

Fasting blood glucose

□more than once a month □more than twice a year □once a year □not routinely

Hemoglobin A1c

□more than once a month □more than twice a year □once a year □not routinely

Dietary habit

□more than once a month □more than twice a year □once a year □not routinely Intake of soft drinks

□more than once a month □more than twice a year □once a year □not routinely

Blood pressure

□more than once a month □more than twice a year □once a year □not routinely

Lipid profile

□more than once a month □more than twice a year □once a year □not routinely

Electrocardiography

□more than once a month □more than twice a year □once a year □not routinely

For outpatients

Fasting blood glucose

□more than once a month □more than twice a year □once a year □not routinely

Hemoglobin A1c

□more than once a month □more than twice a year □once a year □not routinely

Dietary habit

□more than once a month □more than twice a year □once a year □not routinely Intake of soft drink

□more than once a month □more than twice a year □once a year □not routinely

Blood pressure

□more than once a month □more than twice a year □once a year □not routinely

Lipid profile

□more than once a month □more than twice a year □once a year □not routinely

Electrocardiography

□more than once a month □more than twice a year □once a year □not routinely

Q15 How do you decide your frequency of monitoring patients under antipsychotic treatment?

□ own clinical experience

□ guidelines

□ advice of specialists

□ not based on specific principles

Q16 Have you used Homeostasis model assessment-Insulin Resistance (HOMA-IR) in the clinical setting?

□ have not used this definition at all

□ use the definition in some cases

□ usually use the definition

Q17 Do you feel that your frequency of monitoring patients is sufficient?

□ Yes

□ I don’t know

□ No

Q18 Regarding the opportunity to consult with diabetes specialists

□ I can contact a specialist if necessary

□ I usually contact a specialist

□ I cannot contact a specialist

Q19 Regarding the criteria of blood pressure control, I used the definition of

□130/85 □ 140/90 □ 160/10 □other
